# Asthma Management-Induced Diabetic Ketoacidosis: A Case Report

**DOI:** 10.7759/cureus.94894

**Published:** 2025-10-18

**Authors:** Alyaa M Adam, Abdulrahman Y Alzahrani, Elaf Banoon

**Affiliations:** 1 Emergency Department, National Guard Health Affairs, Jeddah, SAU

**Keywords:** asthma management, asthma management induced dka, persistent tachycardia, precipitating factors of dka, steroid-induced dka

## Abstract

Diabetic ketoacidosis (DKA) is an acute, life‑threatening complication of diabetes mellitus, often triggered by infection, missed insulin doses, or physiological stress. Less commonly, medications such as glucocorticoids and repeated short‑acting beta‑agonists (SABAs) may precipitate DKA by exacerbating insulin resistance and promoting gluconeogenesis. We describe a 50‑year‑old diabetic woman who presented during the Hajj season with acute respiratory distress, wheezing, and tachycardia and was initially treated as an acute asthma exacerbation with four consecutive nebulized salbutamol/ipratropium and a methylprednisolone 125 mg IV. Although her breathing improved, persistent tachycardia and progressive hyperglycemia led to further work‑up. She had missed her insulin injections for two days prior to admission. Venous blood gas analysis shortly thereafter revealed a pH of 7.21, elevated lactate, and severe hyperglycemia (random glucose >500 mg/dL), confirming DKA. Initiation of standard DKA management (insulin IV, fluid resuscitation, and electrolyte replacement) resulted in stabilization.

This case highlights the risk of rapid metabolic decompensation in vulnerable patients even after a single dose of corticosteroids combined with high-dose SABA therapy and insulin omission. Atypical presentation in early DKA may include respiratory alkalosis and wheezing, which can delay diagnosis. Clinicians must maintain a high index of suspicion for DKA in diabetic patients presenting with unexplained tachycardia, respiratory symptoms, or gastrointestinal signs, especially when corticosteroids or SABAs are administered. Early recognition and prompt initiation of appropriate therapy are essential to prevent morbidity and improve outcomes.

## Introduction

Diabetic ketoacidosis (DKA) is an acute, life-threatening complication of diabetes mellitus. It results from an absolute or relative insulin deficiency combined with increased levels of counter-regulatory hormones, including glucagon, cortisol, catecholamines, and growth hormone [1]. Typical triggers of DKA include infection, missed insulin doses, and acute illness; however, medications such as glucocorticoids can also precipitate DKA, especially in vulnerable individuals [2]. Corticosteroids, including methylprednisolone, are widely used for their potent anti-inflammatory and immunosuppressive effects. However, they can significantly disrupt glucose metabolism by increasing insulin resistance, enhancing hepatic gluconeogenesis, and impairing peripheral glucose uptake [3]. Especially for those with suboptimal glycemic control or increased physiological stress, this can lead to rapid metabolic decompensation [4]. We report a case of a 50-year-old woman who presented with respiratory symptoms initially treated as asthma. The administration of nebulized salbutamol 5 mg + ipratropium 0.5 mg (4 sets) and IV methylprednisolone contributed to the development of DKA, which was diagnosed only after ward admission due to persistent tachycardia and rising blood glucose levels.

## Case presentation

A 50-year-old woman with a language barrier presented to Alharma Emergency Hospital during the Hajj season with a two-hour history of sudden-onset shortness of breath associated with palpitations. She denied chest pain, paroxysmal nocturnal dyspnea, orthopnea, fever, cough, sore throat, abdominal pain, nausea, vomiting, altered mental status, or urinary symptoms. This was her first such episode.

On examination, the patient appeared anxious, tachypneic, and in respiratory distress, with the use of accessory muscles and audible wheezing. Her random blood sugar was 186 mg/dL. Vital signs on arrival were as follows: blood pressure 130/71 mmHg, heart rate 130 bpm, respiratory rate 28 breaths/min, oxygen saturation 99% on room air, and temperature 37°C. Chest examination revealed decreased bilateral air entry with no added sounds. Cardiovascular examination demonstrated normal S1 and S2 with no additional sounds, the abdomen was soft and non-tender, and the neurological exam was unremarkable.

Initial venous blood gas (VBG) analysis revealed respiratory alkalosis with hypoxemia (pH 7.484, pCO₂ 30.7 mmHg, pO₂ 25.6 mmHg, HCO₃⁻ 22.6 mmol/L, base excess -0.1 mmol/L). Co-oximetry showed a total hemoglobin of 12.6 g/dL, oxyhemoglobin 51.2% (low), and deoxyhemoglobin 48.0% (elevated). Laboratory results are summarized in Table 1.

**Table 1:** Laboratory results BUN: blood urea nitrogen; WBC: white blood cell count; Hb: hemoglobin; AST: aspartate aminotransferase; ALT: alanine aminotransferase; ALP: alkaline phosphatase; CK-MB: creatine kinase-myocardial band

Test	Result	Normal Range	Interpretation
Sodium (Na⁺)	140 mmol/L	135–145 mmol/L	Normal
Potassium (K⁺)	5.0 mmol/L	3.5–5.1 mmol/L	Normal
Creatinine	159 µmol/L	45–90 µmol/L (female)	Elevated
BUN	6.4 mmol/L	2.5–7.1 mmol/L	Normal
Glucose (initial)	180 mg/dL	70–140 mg/dL	Elevated
Glucose (later)	500 mg/dL	70–140 mg/dL	Critically elevated
Lactate (initial)	2.77 mmol/L	0.5–2.2 mmol/L	Mildly elevated
Lactate (later)	10.09 mmol/L	0.5–2.2 mmol/L	Severe elevation
WBC	12.62 ×10⁹/L	4–11 ×10⁹/L	Elevated
Hb	11.9 g/dL	12–16 g/dL	Slightly low
Platelets	377 ×10⁹/L	150–400 ×10⁹/L	Normal
AST	30 U/L	<40 U/L	Normal
ALT	24 U/L	<40 U/L	Normal
ALP	28 U/L	44–147 U/L	Low (not significant)
Albumin	41 g/L	35–50 g/L	Normal
Troponin	0.010 ng/mL	<0.04 ng/mL	Normal
CK-MB	23 U/L	<25 U/L	Normal

ECG demonstrated sinus tachycardia with no ischemic changes. Bedside ultrasonography showed a good ejection fraction, no regional wall motion abnormalities, no pericardial effusion, no right ventricular strain, and a non-collapsing and non-plethoric inferior vena cava. Unfortunately, the original ECG and echocardiography images were not available for inclusion; however, both were assessed and documented by the on-duty consultants from Emergency Medicine and Intensive Care.

The initial impression was acute asthma exacerbation. The patient received back-to-back nebulizations with salbutamol and ipratropium, intravenous methylprednisolone 125 mg, magnesium sulfate 2 g over 30 minutes, and an initial 500 mL bolus of normal saline. Despite clinical improvement in dyspnea, tachycardia persisted (125-130 bpm), prompting an additional 500 mL bolus of fluids. A portable chest radiograph was unremarkable, showing no hyperinflation, infiltrates, or consolidation.

Two hours later, the patient developed heartburn and nausea, for which she was given intravenous omeprazole 40 mg, paracetamol 1 g, and a further 500 mL of normal saline. Repeat cardiac markers and a D-dimer were obtained, both of which were unremarkable. A repeat ECG showed no new changes.

During observation, the patient’s random blood glucose was found to be 500 mg/dL. A repeat VBG test revealed metabolic acidosis (pH 7.211, HCO₃⁻ 10.0 mmol/L, lactate 10.09 mmol/L). The patient was diagnosed with DKA and was immediately started on the hospital’s DKA protocol. The patient was admitted to the intensive care unit for close monitoring. She received intravenous insulin infusion, fluid resuscitation, and electrolyte replacement according to the hospital’s DKA protocol. Over the following hours, her metabolic parameters improved, her dyspnea and tachycardia resolved, and she became hemodynamically stable. She was discharged the next day in good condition, with stable vital signs and no recurrence of respiratory distress or palpitations, and was advised to continue on her own medications with outpatient follow-up.

## Discussion

DKA is a serious and potentially life-threatening complication of diabetes mellitus, commonly precipitated by infection, missed insulin doses, or other forms of physiological stress. Less commonly, glucocorticoids, such as methylprednisolone, can also trigger DKA by exacerbating hyperglycemia and insulin resistance [1,2].

In this case, the patient presented with acute dyspnea, wheezing, and respiratory alkalosis, initially managed as an asthma exacerbation. However, she remained tachycardic despite symptomatic improvement, which prompted medical consultation. Upon further evaluation, she was diagnosed with severe DKA. An important contributing factor, in this case, was the patient translator's report that she had missed her insulin injections for two consecutive days prior to the presentation. This insulin omission likely created a background of subclinical insulin deficiency, and the administration of IV methylprednisolone (125 mg) further tipped the metabolic balance, precipitating DKA.

Additionally, the patient received four back-to-back doses of nebulized short-acting beta-agonists (SABA), salbutamol, and ipratropium as part of the initial management for presumed asthma. High doses or repeated administration of SABA are known to worsen glycemic control by promoting glycogenolysis, gluconeogenesis, and lipolysis, leading to increased production of free fatty acids and ketone bodies. This metabolic shift can aggravate or precipitate DKA in insulin-deficient patients [5].

In addition to insulin omission and medication-related effects, physiological stress plays a significant role in precipitating DKA. Stressful events such as acute illness, infections, or severe asthma exacerbations trigger a surge in counter-regulatory hormones - including catecholamines, cortisol, glucagon, and growth hormone - which antagonize the effects of insulin. These hormones enhance hepatic gluconeogenesis and glycogenolysis while simultaneously impairing peripheral glucose uptake, leading to marked hyperglycemia. Furthermore, stress-induced lipolysis increases the delivery of free fatty acids to the liver, fueling ketone body production and contributing to metabolic acidosis [5].

Corticosteroids are known to promote hepatic gluconeogenesis, decrease peripheral glucose uptake, and increase insulin resistance, all of which impair glycemic control in patients with diabetes [3,6]. When superimposed on missed insulin doses, this can rapidly trigger DKA. Of note, the initial VBG test showed respiratory alkalosis (pH 7.48, HCO₃⁻ 22, pCO₂ 30), which can be seen in the early stages of DKA as part of a compensatory mechanism [4]. This atypical acid-base pattern may have contributed to the initial misdiagnosis.

In addition, the patient's persistent sinus tachycardia was an early but nonspecific clue to underlying metabolic disturbance. Although glucocorticoid-induced DKA is well-described in the context of chronic use or high cumulative doses [7,8], this case illustrates that even a single dose of methylprednisolone can precipitate DKA in a susceptible individual, particularly when compounded by insulin omission.

This case emphasizes the need for heightened awareness when prescribing corticosteroids or beta-agonists to diabetic patients and reinforces the importance of checking serum glucose and ketones in any diabetic patient presenting with unexplained tachycardia, abdominal pain, or new-onset GI symptoms, even if initial acid-base status appears normal.

## Conclusions

This case highlights the importance of maintaining a high index of suspicion for DKA in patients who present with atypical symptoms such as wheezing, respiratory alkalosis, or persistent tachycardia. While missed insulin doses remain a well-established risk factor for DKA, additional contributors - including corticosteroid administration and repeated SABA use - can further accelerate metabolic decompensation. In this case, a single dose of IV methylprednisolone and multiple back-to-back doses of SABA, in the context of two days of missed insulin with a stressor, contributed to the rapid development of severe DKA. Early respiratory alkalosis may have masked the underlying metabolic derangement and delayed appropriate diagnosis. Clinicians should be vigilant when managing diabetic patients with respiratory symptoms and consider early screening for DKA, especially if corticosteroids or beta-agonists are administered. Prompt recognition and treatment are essential to prevent serious complications and improve outcomes.

## References

[1] Kitabchi AE, Umpierrez GE, Miles JM, Fisher JN (2009). Hyperglycemic crises in adult patients with diabetes. Diabetes Care.

[2] Shah P, Kalra S, Yadav Y (2022). Management of glucocorticoid-induced hyperglycemia. Diabetes Metab Syndr Obes.

[3] Gulliford MC, Charlton J, Latinovic R (2006). Risk of diabetes associated with prescribed glucocorticoids in a large population. Diabetes Care.

[4] Nyenwe EA, Kitabchi AE (2016). The evolution of diabetic ketoacidosis: An update of its etiology, pathogenesis and management. Metabolism.

[5] Cowie CC, Casagrande SS, Menke A (2018). Diabetes in America, 3rd ed. Diabetes in America. 3rd ed. Bethesda (MD): National Institute of Diabetes and Digestive and Kidney Diseases (US).

[6] van Raalte DH, Diamant M (2014). Steroid diabetes: from mechanism to treatment?. Neth J Med.

[7] Hwang JL, Weiss RE (2014). Steroid-induced diabetes: a clinical and molecular approach to understanding and treatment. Diabetes Metab Res Rev.

[8] Cavataio MM, Packer CD (2022). Steroid-induced diabetic ketoacidosis: A case report and review of the literature. Cureus.

